# Associations between *SLC16A11* variants and diabetes in the Hispanic Community Health Study/Study of Latinos (HCHS/SOL)

**DOI:** 10.1038/s41598-018-35707-7

**Published:** 2019-01-29

**Authors:** Bertha A. Hidalgo, Tamar Sofer, Qibin Qi, Neil Schneiderman, Y.-D. Ida Chen, Robert C. Kaplan, M. Larissa Avilés-Santa, Kari E. North, Donna K. Arnett, Adam Szpiro, Jianwen Cai, Bing Yu, Eric Boerwinkle, George Papanicolaou, Cathy C. Laurie, Jerome I. Rotter, Adrienne M. Stilp

**Affiliations:** 1 University of Alabama at Birmingham, Department of Epidemiology, Birmingham, Alabama USA; 2 Brigham and Women’s Hospital, Harvard Medical School, Boston, Massachusetts USA; 3 Albert Einstein College of Medicine, Department of Epidemiology and Population Health, Bronx, New York USA; 4 University of Miami, Department of Psychology and Behavioral Medicine Research Center, Miami, Florida USA; 5 Institute for Translational Genomics and Population Sciences, Los Angeles Biomedical Research Institute and Department of Pediatrics at Harbor-UCLA Medical Center, Los Angeles, California USA; 6 National Institutes of Health, National Heart, Lung, and Blood Institute, Bethesda, Maryland USA; 7 University of Chapel Hill, Department of Epidemiology, Chapel Hill, North Carolina USA; 8 University of Kentucky, College of Public Health, Lexington, Kentucky USA; 9 University of Washington, Seattle, Department of Biostatistics, Seattle, Washington USA; 10 University of North Carolina, Chapel Hill, Department of Biostatistics, Chapel Hill, North Carolina USA; 11 Public Health Sciences Division, Fred Hutchinson Cancer Research Center, Seattle, Washington USA; 12 University of Texas, Health Science Center, Houston, Texas USA

## Abstract

Five sequence variants in *SLC16A11* (rs117767867, rs13342692, rs13342232, rs75418188, and rs75493593), which occur in two non-reference haplotypes, were recently shown to be associated with diabetes in Mexicans from the SIGMA consortium. We aimed to determine whether these previous findings would replicate in the HCHS/SOL Mexican origin group and whether genotypic effects were similar in other HCHS/SOL groups. We analyzed these five variants in 2492 diabetes cases and 5236 controls from the Hispanic Community Health Study/Study of Latinos (HCHS/SOL), which includes U.S. participants from six diverse background groups (Mainland groups: Mexican, Central American, and South American; and Caribbean groups: Puerto Rican, Cuban, and Dominican). We estimated the SNP-diabetes association in the six groups and in the combined sample. We found that the risk alleles occur in two non-reference haplotypes in HCHS/SOL, as in the SIGMA Mexicans. The haplotype frequencies were very similar between SIGMA Mexicans and the HCHS/SOL Mainland groups, but different in the Caribbean groups. The *SLC16A11* sequence variants were significantly associated with risk for diabetes in the Mexican origin group (P = 0.025), replicating the SIGMA findings. However, these variants were not significantly associated with diabetes in a combined analysis of all groups, although the power to detect such effects was 85% (assuming homogeneity of effects among the groups). Additional analyses performed separately in each of the five non-Mexican origin groups were not significant. We also analyzed (1) exclusion of young controls and, (2) SNP by BMI interactions, but neither was significant in the HCHS/SOL data. The previously reported effects of *SLC16A11* variants on diabetes in Mexican samples was replicated in a large Mexican-American sample, but these effects were not significant in five non-Mexican Hispanic/Latino groups sampled from U.S. populations. Lack of replication in the HCHS/SOL non-Mexicans, and in the entire HCHS/SOL sample combined may represent underlying genetic heterogeneity. These results indicate a need for future genetic research to consider heterogeneity of the Hispanic/Latino population in the assessment of disease risk, but add to the evidence suggesting *SLC16A11* as a potential therapeutic target for type 2 diabetes.

## Introduction

Hispanics/Latinos represent the largest ethnic minority population in the United States^1^. They are a diverse group of individuals, varying greatly from one another genetically, socially, economically, and culturally, despite usually being classified as a single ethnic group. In particular, variation in the prevalence of diabetes among Hispanic/Latino groups^2^ indicates that specific Hispanic/Latino background should be considered in genetic and other analyses.

*SLC16A11* is a member of the solute carrier family 16, which appears to be involved in hepatic lipid metabolism^3^. Williams *et al*. reported an *SLC16A11* haplotype, defined by 5 single nucleotide polymorphisms (SNPs), as a common risk factor for diabetes in Mexican and Mexican-American populations studied by the SIGMA consortium^3^. Four of the five variants are missense SNPs, and the frequency of the risk haplotype is high (~50%) in Hispanics/Latinos with high Native American ancestry but rare or absent in people of European and African ancestry. Results from the discovery sample were replicated in a meta-analysis of several multi-ethnic populations, in which most of the evidence appeared to come from Native Hawaiian, East Asian and Mexican American samples.

Here, we examined the SNP associations with diabetes reported by Williams *et al*., in U.S. Hispanics/Latinos from the Hispanic Community Health Study/Study of Latinos (HCHS/SOL), which includes individuals who self-identified as having Mexican, Central American, South American, Puerto Rican, Dominican or Cuban background or heritage (Table 1). We assessed whether the associations of the five SNPs with diabetes status were observed in each of these groups and whether there is evidence of group-specific effects. In addition, we tested these five SNPs for interaction with obesity in their effects on diabetes to test associations described by Traurig *et al*.^4^, as described below.

**Table 1:** Demographics for cases and controls in HCHS/SOL.

Group		N	Age (SD)	Male, %	BMI (SD)	Fasting glucose (SD)
All	Controls	5236	38.9 (13.4)	39.6	28.1 (5.6)	89.7 (5.7)
	Cases	2492	55.0 (10.3)	39.8	31.2 (6.5)	149.2 (64.0)
Dominican	Controls	523	37.3 (13.4)	32.9	28.1 (5.5)	88.7 (5.7)
	Cases	217	55.9 (10.3)	35.0	31.3 (6.2)	139.8 (57.0)
Mexican	Controls	1914	37.9 (13.5)	38.5	28.1 (5.4)	89.6 (5.7)
	Cases	965	52.8 (10.7)	39.1	32.0 (6.2)	151.5 (66.0)
Puerto Rican	Controls	840	38.9 (13.9)	42.4	28.9 (6.5)	89.0 (5.9)
	Cases	557	56.7 (10.2)	39.3	33.4 (7.1)	148.6 (61.6)
Cuban	Controls	918	42.3 (13.3)	41.5	27.7 (5.5)	90.3 (5.7)
	Cases	396	57.9 (9.2)	47.2	31.7 (6.2)	149.9 (65.9)
South American	Controls	450	40.0 (12.8)	39.6	27.1 (4.6)	90.1 (5.7)
	Cases	113	56.2 (9.2)	34.5	31.5 (6.1)	137.5 (54.2)
Central American	Controls	591	37.9 (12.5)	42.0	28.4 (5.4)	90.3 (5.4)
	Cases	244	54.2 (9.2)	38.9	32.1 (6.1)	154.3 (67.6)

## Methods

### Study Sample

HCHS/SOL is a multicenter community–based cohort study of Hispanic/Latino populations in the United States, previously described^5,6^. Of the 12,803 individuals successfully genotyped in SOL, 428 did not self-identify as one of the six specific background groups. The 428 exceptions either had missing, multiple or ‘other’ background. These 428 individuals were not outliers with respect to the entire sample set. PCs indicated that some individuals were outliers with respect to their self-identified group, but not with respect to other backgrounds. Therefore, six ‘genetic analysis groups’ using both self-identified background and principal components so that all individuals would be included in the specific background group and to improve the genetic homogeneity within those groups, were included in these analyses. The analyses described here therefore included 2,492 individuals with diabetes and 5,236 controls, for a total of 7,728 individuals, with demographic and diabetic characteristics shown in Table 1. The study was conducted with the approval of the Ethics and Institutional Review Boards of all institutions involved (i.e., Bronx Field Center – Albert Einstein School of Medicine; Chicago Field Center – University of Illinois Chicago; Miami Field Center – University of Miami; San Diego Field Center – San Diego State University), and informed consent was obtained from all participants. HCHS/SOL was conducted under the oversight of each institutional review board (IRB) at the field centers and coordinating center institutions, http://www.cscc.unc.edu/hchs. HCHS/SOL had an Observational Studies Monitoring Board that served as advisory to the NHLBI and provided oversight on participant burden, safety, study progress. Further, all methods were performed in accordance with the relevant guidelines and regulations.

### Definition of Diabetic Status

In accordance with the American Diabetes Association (ADA)^7^, individuals with diabetes were defined as those with fasting time >8 hours and fasting glucose levels ≥126 mg/dL; or fasting ≤8 hours and fasting glucose ≥200 mg/dL; or post-oral glucose tolerance test (OGTT) glucose ≥200 mg/dL; or hemoglobin A1C (HbA1C) ≥6.5%; or if on current treatment with a hypoglycemic agent. Controls with normal glucose tolerance were defined as anyone with fasting time >8 hours and fasting glucose levels less than 100 mg/dL; and post-OGTT glucose less than 140 mg/dL; and HbA1C less than 5.6%. Individuals with pre-diabetes intermediate phenotypes were excluded from this analysis. We were unable to cleanly separate T2D from T1D for the sample included in this analysis because T1D is largely an autoimmune disease identified by at least one diabetes autoantibody (glutamic acid decarboxylase or insulinoma associated antibody)^8^ and these measures were not assessed in HCHS/SOL. Furthermore, studies show that the age of onset of T2D has substantially decreased in the last few years, so that age at diagnosis could not be used to distinguish between diabetes types^9,10^. In any case, it seems unlikely that results will be affected substantially by not making the type 1 versus type 2 distinction, given that within our HCHS/SOL sample, only 9 individuals in our sample aged 18–29 years could potentially have T1D based on use of insulin^2^ and all participants used in this analysis were greater than 18 years of age. Among those, 3 were Dominican, 1 Central American, 4 Mexican, and 1 Puerto Rican. Finally, the prevalence of T1D in the United States was estimated to be only 4.3% in 2012, further indicating the low prevalence of individuals with type 1 diabetes in the general population^7^.

### Genotyping and imputation

Genotyping was performed with an Illumina custom array (15041502 B3), which consists of the Illumina Omni 2.5 M array (HumanOmni2.5-8v1-1) plus approximately 150k custom SNPs. QA/QC methods have been previously described^11,12^. Genome-wide imputation was carried out using the 1000 Genomes Project phase 1 reference panel^13^, SHAPEIT2^12^ and IMPUTE2 software^14^, as described previously^12^.

### Relatedness, population structure, and genetic analysis groups

Kinship coefficients and principal components were estimated using PC-Relate^12^. Genetic analysis groups were constructed based on a combination of self-identified Hispanic/Latino background and genetic similarity, and are classified as Cuban, Dominican, and Puerto Rican (Caribbean groups); and Mexican, Central American, and South American (Mainland groups). The genetic analysis groups largely overlap with the self-identified background groups, but using the genetic analysis groups in association testing and stratified analyses has advantages as shown by Conomos *et al*.^12^. Briefly, Conomos *et al*., showed that using genetic analysis group (as we did in this analysis and manuscript), rather than a self-identified background group “achieved higher power to detect previously reported associations”. The average proportions of three continental ancestries (European, African and Native American) differ among these groups, with Caribbean groups having more African and less Native American ancestry than the Mainland groups^12^.

### Haplotype frequency estimation

**Figure 1:**
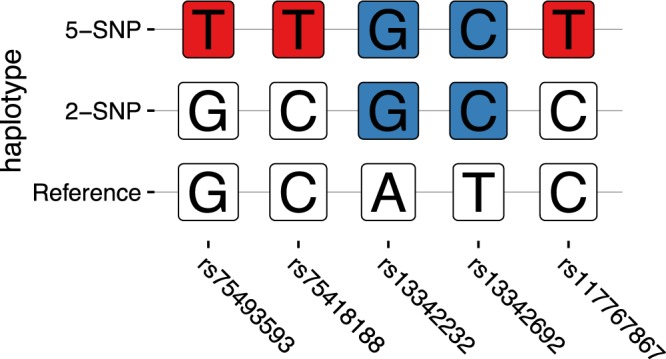
SLC16A11 haplotypes in HCHS/SOL. The lower panel shows the reference haplotypes, with the reference (major) alleles for all 5 SNPs. The 2-SNP haplotype is composed of the non-reference (minor) alleles of the two blue SNPs, with the reference alleles for the other three SNPs. The 5-SNP haplotype has non-reference alleles for all 5 SNPs. The blue SNPs are LD group 1, and the red SNPs are LD group 2.

Genotypes for the five SNPs constituting the risk haplotype defined by the SIGMA consortium are either assayed on the array or very well imputed (imputation “info” score >0.99) in HCHS/SOL (Table 2). To confirm imputation quality, we also performed both a Spearman correlation analysis as well as a genotype comparison between the data used in this analysis and the HCHS/SOL whole genome sequence (WGS) data, wherein we found that the concordance between the two platforms was high – all SNPS had a correlation coefficient greater than 0.99. There were only a handful of mismatches between SNPs measured and those imputed, which even if confined to one group, would not represent substantial inaccuracy in imputation. In HCHS/SOL, these five SNPs formed the same three haplotypes as seen in the Williams *et al*. study (Fig. 1). The minor alleles of the five SNPs may appear together to form the 5-SNP haplotype, or the minor alleles of only two of the SNPs (rs13342232 and rs13342692; “LD group 1”) may appear with the reference alleles of the other three SNPs (rs75493593, rs75418188, and rs117767867; “LD group 2”) to form the 2-SNP haplotype. The SNPs within each of the two LD groups are highly correlated (r2 > 0.99). Therefore, we estimated haplotype frequencies as in Williams *et al*.^3^ the 5-SNP haplotype as the frequency of the minor allele of a given SNP from LD group 2, and the frequency of the 2-SNP haplotype as the frequency of the major allele of a given SNP from LD group 1 minus the frequency of a given SNP from the 5-SNP haplotype.

**Table 2:** Haplotype structure of five coding sequence variants in the SLC16A11 gene and their estimated frequencies.

SNP	Inferred haplotypes			chr17: position	Amino Acid	LD group*	Type
	Reference	2-SNP	5-SNP				
rs75493593	G	G	T	6945087	P443T	2	Imputed (r2 = 99.9)
rs75418188	C	C	T	6945483	G340S	2	Imputed (r2 = 99.9)
rs13342232	A	G	G	6945940	L187L	1	genotyped
rs13342692	T	C	C	6946287	D127G	1	genotyped
rs117767867	C	C	T	6946330	V113I	2	Imputed (r2 = 99.8)

*LD group: linkage disequilibrium between SNP pairs within each of the two groups in HCHS/SOL is r^2^ > 0.99, while r^2^ values for SNP pairs between groups varies among groups (0.75 for Central American, 0.89 for Mexican, 0.85 for South American, 0.37 for Cuban, 0.11 for Dominican and 0.33 for South American groups).

### Power analysis

We calculated power for replicating the association reported by Williams *et al*.^3^ for rs75493593. We considered replication in the Mexican group, other individual groups, and the combined analysis of all HCHS/SOL analysis participants. To avoid a potential bias in the odds ratio (OR) estimation due to the winner’s curse, we used OR = 1.20 (95%CI = 1.09–1.31) as estimated in the replication study of Williams *et al*., rather than the discovery estimate of OR = 1.29. We calculated power following^15^, assuming a significance level of 0.025 (for testing two haplotypes), as described further in Supplementary Material.

### Statistical analysis

Our association analyses focused on the 5-SNPs haplotype reported in Williams *et al*. Because this haplotype is tagged by the minor allele of rs75493593, we report association analyses results for this SNP. Rs75493593-diabetes association analysis was performed using GMMAT^16^, which is based on a logistic penalized quasi-likelihood (PQL) model that approximates the logistic generalized linear mixed model. Correlations between the HCHS/SOL participants were accounted for by incorporating covariance matrices corresponding to genetic relatedness (kinship), household, and census block group as random effects. The model included center, age, sex, log10 BMI, the first five principal components to adjust for ancestry, and sampling weights^17^. To study how diabetes associations with rs75493593 vary by genetic analysis group, we included statistical interaction terms in the model. The PQL also estimated the covariance between the group-specific effect estimates. We then obtained pooled estimates of the genotype effect estimates, as well as the Cochran Q test of heterogeneity, using MetaCor^18^, which accounts for correlations between group-specific effect estimates (see Supplementary Material). Results for each of the specific SNPs in the haplotype are provided in the Supplementary Material.

## Results

### SLC16A11 SNPs and Haplotypes

While the haplotype structure reported in Williams *et al*.^3^ also exists in the HCHS/SOL, the haplotype frequencies varied across genetic analysis groups (Table 3). In the Mexican, Central and South American groups, the haplotype frequencies were similar to those in the SIGMA Mexicans. In the three Caribbean groups, the frequencies of the 5-SNP haplotype were substantially lower than in the three Mainland groups, as expected because this haplotype appears to be largely specific to Amerindian ancestry, which is low in Caribbean groups^12^. In addition, the 2-SNP haplotype, which is largely specific to African ancestry, occurred at higher frequency in the Caribbean than in the Mainland groups, as expected because African ancestry is low in Mainland groups^12^.

**Table 3:** Estimated frequencies of inferred haplotypes.

		Haplotype frequency estimates**		
		Reference	2-SNP	5-SNP
SIGMA	Mexican	0.68	0.02	0.30
HCHS/SOL	Mexican	0.70	0.02	0.28
	Central American	0.73	0.05	0.22
	South American	0.73	0.03	0.24
	Cuban	0.90	0.06	0.04
	Dominican	0.80	0.17	0.03
	Puerto Rican	0.82	0.11	0.07
1000G phase 3	AFR	0.62	0.38	0.00
	AMR	0.72	0.03	0.24
	EAS	0.90	0.00	0.10
	EUR	0.98	0.01	0.01
	SAS	0.99	0.00	0.00

**Haplotype frequency estimates for HCHS/SOL were derived from unphased genotypes (as described in Methods); for 1000 G, they were calculated directly from phased genotypes. Listed in same order as haplotypes above.

### Rs75493593 *Associations with Diabetes Status*

**Figure 2:**
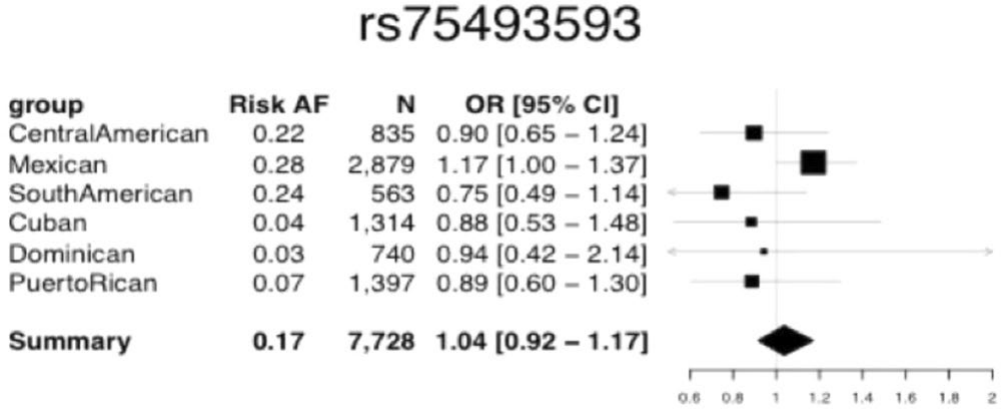
Summary results for association analysis of rs75493593, which tags the 5-SNP haplotype, with diabetes in the HCHS/SOL. Odds ratio estimates and their 95% confidence intervals are given in the Forest plots. Risk “AF” refers to risk allele frequency. “Summary” gives meta-analysis results. The meta-analysis replication (one-sided) p-value was 0.28, while the replication p-value in Mexicans was 0.025.

The expected power to replicate the 5-SNP haplotype, tagged by rs75493593, effect on diabetes status was 0.85 for all groups combined, 0.55 for the Mexican group, and 0.04 to 0.14 for each of the other genetic analysis groups (assuming a homogeneous effect with OR = 1.20; see Supplementary Material). The SNP effect estimates for the Mexican group were all directionally consistent with those reported in the Williams’ paper, and four of the five SNPs replicated (one-sided p = 0.025; Supplementary Material). The odds ratio estimate for the top SNP rs75493593 in the HCHS/SOL Mexican group was 1.17 (CI: 1.00–1.37, p = 0.025), compared with OR = 1.29 (95% CI: 1.20–1.38) in the SIGMA discovery and OR = 1.20 (95% CI: 1.09–1.31) in the SIGMA replication set. The effect estimates in each of the non-Mexican groups are in the opposite direction from the effect estimate for the Mexican background group but are not significant, although the test for heterogeneity among the groups is suggestive of possible heterogeneity (p = 0.07; Supplementary Material; Fig. 2). In a meta-analysis of all groups, the association of *SLC16A11* variants with diabetes was not significant (p = 0.27).

We repeated these association tests after excluding controls <45 years old (3436 participants) to better approximate the control definitions used in some of the Williams *et al*. sample sets. The results are qualitatively similar to the full sample set, but no SNPs are statistically significant in any group, likely due to less power from the smaller sample set (see Supplementary Material).

Previously, Traurig *et al*.^4^ reported that the 5-SNP haplotype in a Native North American sample has a significant interaction with obesity, such that the rs75493593 risk allele (marking the 5-SNP haplotype) has a positive effect estimate in individuals with low body mass index (BMI), while having a negative effect estimate in those with high BMI. Such a relationship could explain the apparent heterogeneity in effect estimates among the HCHS/SOL groups if a similar interaction occurs in these populations and if the Mexican group has lower BMI. However, neither one of these conditions was observed (see Supplementary Material).

## Discussion

The initial SIGMA discovery of an association between *SLC16A11* and diabetes was from a GWAS of Mexicans and Mexican-Americans, with replication through meta-analysis of a set of cohorts of diverse ancestries^3^. In HCHS/SOL, we found that the 5-SNP haplotype is significantly associated with diabetes in participants of Mexican background (p = 0.025), with the same direction of effect as in SIGMA. However, the association is not significant in the HCHS/SOL cohort as a whole (despite 85% power to detect a significant effect), nor is it significant within any of the other five Hispanic/Latino background groups. We also observed that the 95% confidence intervals in each of the subgroups include the point estimate for the positive association in Mexicans, even if not significant. Thus, while non-replication of the effect in any specific group could be explained by lack of power, the power was high in the combined analysis. In fact, the effect estimates for the five non-Mexican groups are consistently in the opposite direction of the effect in the Mexican group. A test of SNP-by-group interaction has a P-value of 0.07, further suggesting not only the heterogeneity of effect among these diverse Hispanic/Latino groups, but providing further evidence of the specificity of *SLC16A11* in Mexican-origin populations.

The estimated effects between the HCHS/SOL Mexicans and the other groups is unexpected, given that allelic and haplotypic frequencies are very similar among the HCHS/SOL Central American, South American and Mexican groups. Furthermore, the Williams *et al*. study generalized their initial finding in Mexicans to diverse populations, including East Asians. One might expect that a finding in Mexicans that generalizes to East Asians, should also generalize to other Hispanic/Latino populations more similar to Mexicans, such as Central and South Americans. We further hypothesized that this apparent heterogeneity among HCHS/SOL groups might be caused by differing age or BMI distributions, but the results of these analyses did not reveal more insights (see Supplementary Material). We speculate that the variation among groups might be due to variation in pattern of LD with the causal variant(s), interactions with other genetic variants that differentiate the groups, or with non-genetic differences among the groups. It is unlikely that an LD plot of this region would not have provided much clarity, or that it would have demonstrated a significant difference between groups. Another possibility is simply that the predicted high power to detect an overall effect in the HCHS/SOL cohort was not realized due to un-modeled sources of variability or residual confounding.

In this study, we chose to limit our analyses to the *SLC16A11* variants, however have explored other T2D-associated variants in HCHS/SOL elsewhere^19^. Other studies have also examined the replication of the Williams *et al*. result in related populations. Traurig, *et al*. also found that the 5-SNP haplotype is significantly associated with diabetes in a sample of 12,811 Native North Americans, with an effect dependent on BMI^4^. Others have investigated the role of one of the five variants in a sample of 575 Mayan individuals from Mexico, finding that rs13342692 was not significantly associated with diabetes after adjustment for BMI^20^, but given the small sample the lack of replication may be due simply to low power. Recent *SLC16A11* functional work in individuals of Mexican origin by Rusu, *et al*. suggests that T2D disrupts gene function at this locus and could be a therapeutic target for this population. We performed an LD analysis of these new sequence variants in our data and found that the LD between the 5 SNPs in our study and the new SNPs in the Rusu *et al*. paper is high (r^2^ > 0.85), as expected. Rusu, *et al*. reported 13 new SNPs, and we discussed the additional 5 SNPs in this paper. Of those, 11 SNPs were in our imputed data and in high LD with the variants in our LD group 2; 8 of them had LD > 0.98 and the other 3 had LD > 0.85, further supporting our Mexican-origin specific findings^21^. Our HCHS/SOL results contribute to understanding the genetic underpinnings of diabetes in Mexicans, indicate a need for future genetic research to consider heterogeneity of the Hispanic/Latino population in the assessment of disease risk, and provides additional evidence suggesting that *SLC16A11* could be a therapeutic target for T2D.

## Electronic supplementary material

Supplementary Information

## Data Availability

The data that support the findings of this study are available from HCHS/SOL but restrictions apply to the availability of these data, which were used under license for the current study, and so are not publicly available. Data are however available from the authors upon reasonable request and with permission of HCHS/SOL Steering Committee.
