# Supplementary material for: Associations between SLC16A11 variants and diabetes in the Hispanic Community Health Study/Study of Latinos (HCHS/SOL)
Source: Sci Rep. 2019 Jan 29;9:843. doi: 10.1038/s41598-018-35707-7 (PMC6351621; doi:10.1038/s41598-018-35707-7)

Associations between SLC16A11 variants and diabetes in the Hispanic Community Health Study/Study of Latinos (HCHS/SOL)
Online supplementary material

Bertha Hidalgo, Tamar Sofer, Qibin Qi, Neil Schneiderman, Y.-D. Ida Chen, Robert Kaplan, Larissa Avilés-Santa, Kari E. North, Donna K. Arnett, Adam Szpir, Jianwen Cai, Bing Yu, Eric Boerwinkle, George Papanicolaou, Cathy C. Lauri, Jerome I. Rotter, Adrienne M. Stilp

October 25, 2018

Contents

1 Statistical model **1**

2 Power analysis **3**

3 Older control analysis **5**

4 BMI and *SLC16A11* effects on Diabetes Status **6**

5 Fine Mapping - *SLC16A11* **9**

6 Diabetes status SNP Effects – Meta analysis Results **11**

1 Statistical model

We performed SNP-DM association analyses using a logistic generalized linear mixed model (GLMM). The GLMM model was defined as

$$\log \frac{p(D=1)}{p(D=0)} = \alpha_0 + X^T\alpha_x + PC^T\alpha_{pc} + gI_{(Mexican)}\alpha_{Mex} + \ldots + gI_{(Cuban)}\alpha_{cub},$$

where $p(D = 1)$ and $p(D = 0)$ are the probabilities of being a case and a control, respectively, α_0 is the baseline log-odds of the disease in the study sample, X is a vector of covariates, including study center, age, sex, log BMI, and the sampling weights, and PC is the vector of first 5 genetic principal components to adjust for ancestry. The genetic effects were incorporated as interaction variables between the genotype and each of the genetic analysis groups, defined by $gI_{(\text{genetic analysis group})}$, where $I_{(\text{genetic analysis group})}$ is the indicator function being equal to 1 when a participant belongs to the specific genetic analysis group. The parameters $\alpha_{Mex}, \alpha_{Cub}$, etc, estimate the log odds ratios (ORs) of the genotype on diabetes status in each of the genetic analysis groups. In practice, because some of the HCHS/SOL participants are correlated to each other due to the study design, we fit a logistic penalized quasi-likelihood (PQL, N. E. Breslow (1993)) model that approximates the GLMM while accounting for correlations between the HCHS/SOL participants by incorporating covariance matrices corresponding to genetic relatedness (kinship), household, and community (census block group) as random effects. The PQL and the modeling of the group-specific effects using an interaction model allowed us to retain all eligible participants while approximating a stratified model, whereas a stratification would require discarding some individuals due to the correlations. The PQL also estimated the covariance between the group-specific effects estimates. Let α_g be the vector of six estimated group-specific genotype effects. Denote the variance-covariance of α_g by $\text{cov}(\alpha_g)$. We obtained pooled estimates of the genotype effect estimates using MetaCor, a meta-analysis methodology that accounts for correlations between groups-specific effect estimates (Sofer et al., 2015). Specifically, let 1 be the vector of ones, with the same length as the vector α_g. Then $\alpha_{pooled} = (\mathbf{1}^T \text{cov}(\alpha_g)^{-1}\alpha_g)/(\mathbf{1}^T \text{cov}(\alpha_g)^{-1}\mathbf{1})$, with $\text{var}(\alpha_{pooled}) = \mathbf{1}^T \text{cov}(\alpha_g)^{-1}\mathbf{1}$. When the group-specific effect estimates are uncorrelated, MetaCor becomes the usual inverse-variance meta-analysis estimator. Finally, we also tested for heterogeneity between the group-specific effect estimates using the Cochran Q test, adapted to the correlations between the effect

estimates.

2 Power analysis

We calculated power for detecting the reported association of the risk haplotype with DM in the HCHS/SOL sample sets. Since the frequency of the risk haplotype is approximated by the frequency of SNPs from LD group 1, we based our power calculation on the frequency of rs75493593. We used three OR values. $OR_1 = 1.25$ from the combined discovery + replication data, $OR_2 = 1.20$ from the replication data alone, and $OR_3 = 1.09$, the lower end of the 95% confidence interval for replication reported by Williams et al.

We first converted the provided ORs into risk ratios in the following manner: consider the frequently assumed, logistic, additive, disease probability model, given by:

$$\text{logit}(p(D = 1|g) = \alpha + \beta g$$

Where here α is the log-odds in the people with zero count of the allele g, and β is the log(OR). For any given subset of interest of the HCHS/SOL data (e.g. Mexican, all people, etc.), we first estimate α using all individuals in the group who do not have a copy of the minor allele g (i.e. dot not carry the risk haplotype). For these people, we estimate $p(D = 1|g = 0)$ as the proportion of diseased non-carriers, and we find α using the logit function, i.e.:

$$\alpha = \log\{p(D = 1|g = 0)/[1 - p(D = 1|g = 0)]\} = \text{logit}[p(D = 1|g = 0)].$$

Note here that to estimate the log-odds parameter we used the non-carriers from entire HCHS/SOL genotyped sample set, i.e. without removing pre-diabetics. Therefore, we had 8972 available individuals to estimate α. The inverse function of the logit function, called the "expit" function, is given such that

$$p(D=1|g) = \exp(\alpha + \beta g)/[1 + exp(\alpha + \beta g)] = \text{expit}(\alpha + \beta g).$$

Based on the log-odds parameter α and the definition of the risk ratio, we can estimate the risk ratio in people who carry a single copy, and people who carry two copies, of the risk

haplotype, according to:

$$RR_1 = p(D=1|g=1)/p(D=1|g=0) = expit(\alpha+\beta)/expit(\alpha)$$
$$RR_2 = p(D=1|g=2)/p(D=1|g=0) = expit(\alpha+2\beta)/expit(\alpha).$$

To calculate power we needed risk ratio parameters, numbers of cases and controls, haplotype frequency, and disease prevalence in the population. To calculate disease prevalence, we again included the pre-diabetics so the sample is representative of the population as much as possible. Therefore, the disease prevalence in each of the groups of interest was estimated as the proportion of diseased individuals, regardless of carrier status, and including pre-diabetics.

Finally, based on the risk ratios, the number of cases and controls, using the minor allele frequencies observed in appropriate subsample of the HCHS/SOL and assuming a significance level of 0.025, we followed Gordon et al. (2002) to calculate power. Table S1 provides power for each of the subgroups used for power calculation and each of the ORs.

group	AF	OR =1.25	OR=1.20	OR=1.09
All	0.17	0.96	0.85	0.24
CentralAmerican	0.22	0.21	0.14	0.05
Mexican	0.28	0.74	0.55	0.13
SouthAmerican	0.24	0.14	0.10	0.04
Cuban	0.04	0.08	0.06	0.03
Dominican	0.03	0.05	0.04	0.03
PuertoRican	0.07	0.14	0.10	0.04

Table S1: Estimated replication power for each subset of HCHS/SOL participants, and for multiple OR values.

rs13342232

group	Risk AF	N	OR [95% CI]
CentralAmerican	0.27	434	0.93 [0.67 − 1.30]
Mexican	0.30	1,618	1.16 [0.98 − 1.38]
SouthAmerican	0.27	290	0.78 [0.53 − 1.16]
Cuban	0.10	830	0.80 [0.56 − 1.14]
Dominican	0.20	403	0.93 [0.64 − 1.36]
PuertoRican	0.19	877	0.77 [0.59 − 1.01]
**Summary**	**0.23**	**4,452**	**0.96 [0.86 − 1.08]**

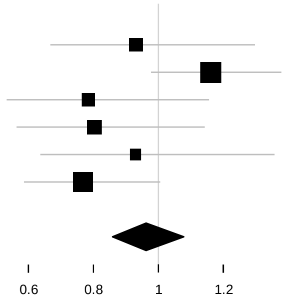

rs75493593

group	Risk AF	N	OR [95% CI]
CentralAmerican	0.21	434	0.93 [0.66 − 1.33]
Mexican	0.28	1,618	1.16 [0.98 − 1.39]
SouthAmerican	0.23	290	0.78 [0.50 − 1.19]
Cuban	0.04	830	0.78 [0.47 − 1.30]
Dominican	0.03	403	0.84 [0.36 − 1.95]
PuertoRican	0.07	877	0.86 [0.57 − 1.29]
**Summary**	**0.16**	**4,452**	**1.02 [0.89 − 1.16]**

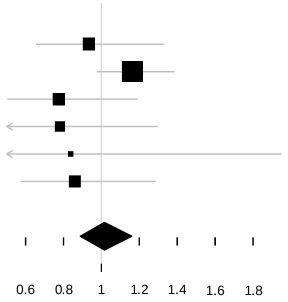

Figure S1: Forest plots of results from an analysis including only controls with age > 45 years.

3 Older control analysis

The diabetes case-control association with SLC16A11 SNPs described in the main text was repeated after excluding controls < 45 years old (3436 participants) to better approximate the control definitions used in some of the Williams et al. sample sets. Figure S1 (compared with Figure 1 in the main text) shows that the results are qualitatively similar to the full sample set, but that no SNPs are statistically significant in any group, likely due to less power from the smaller sample set.

4 BMI and *SLC16A11* effects on Diabetes Status

To determine whether BMI differs between the HCHS/SOL Mexican group and the remaining groups, we regressed BMI on age, sex, center, log sampling weight, and an indicator for being in the Mexican group, using the 7,728 HCHS/SOL participants included in our primary analysis. Random effects for block group, household, and genetic relatedness were also included. BMI was not significantly different between the Mexican and other groups (p=0.57, Figure S2). When we examined the effect of being in the Mexican group vs. not on BMI without adjusting for age, sex, center, and sampling weights, we still observed a large, insignificant p-value for being in the Mexican group (data not shown).

To determine whether the rs75493593 SNP effect on diabetes status varies among BMI categories in HCHS/SOL, as described previously by Traurig et al. for North American Natives, we created a five-level BMI categorical variable ([0,25) N=1829; [25,30) N= 2846, [30,35) N=1850; [35,40) N=747; [40,100) N=456) to match that used in the Traurig study. Diabetes case/control status was regressed on age, sex, center, genetic PCs, log sampling weight, BMI category and SNP × BMI category interaction. The three random effects for block group, household and genetic relatedness were also included. The SNP × BMI category interaction was not significant (p=0.49). See Figure S2 for OR estimates by BMI category.

References

Gordon, D., Finch, S. J., Nothnagel, M. and Ott, J. (2002). Power and sample size calculations for case-control genetic association tests when errors are present: application to single nucleotide polymorphisms. *Human heredity*, **54** 22–33.

N. E. Breslow, D. G. C. (1993). Approximate inference in generalized linear mixed models. *Journal of the American Statistical Association*, **88** 9–25.

Sofer, T., Shaffer, J. R., Graff, M., Qi, Q., Stilp, A. M., Gogarten, S. M., North, K. E., Isasi, C. R., Laurie, C. C. and Szpiro, A. A. (2015). Meta-analysis of genome-wide association studies with correlated individuals: application to the Hispanic

Community Health Study/Study of Latinos (HCHS/SOL). Tech. rep., UW Biostatistics Working Paper Series.

M. Traurig, R.L. Hanson, A. Marinelarena, S. Kobes, P. Piaggi, S. Cole, J.E. Curran, J.Bl angero, H. Göring, S. Kumar, *et al.*Analysis of SLC16A11 Variants in 12,811 American Indians: Genotype-Obesity Interaction for Type 2 Diabetes and an Association With RNASEK Expression Diabetes, 65 (2016), pp. 510-519

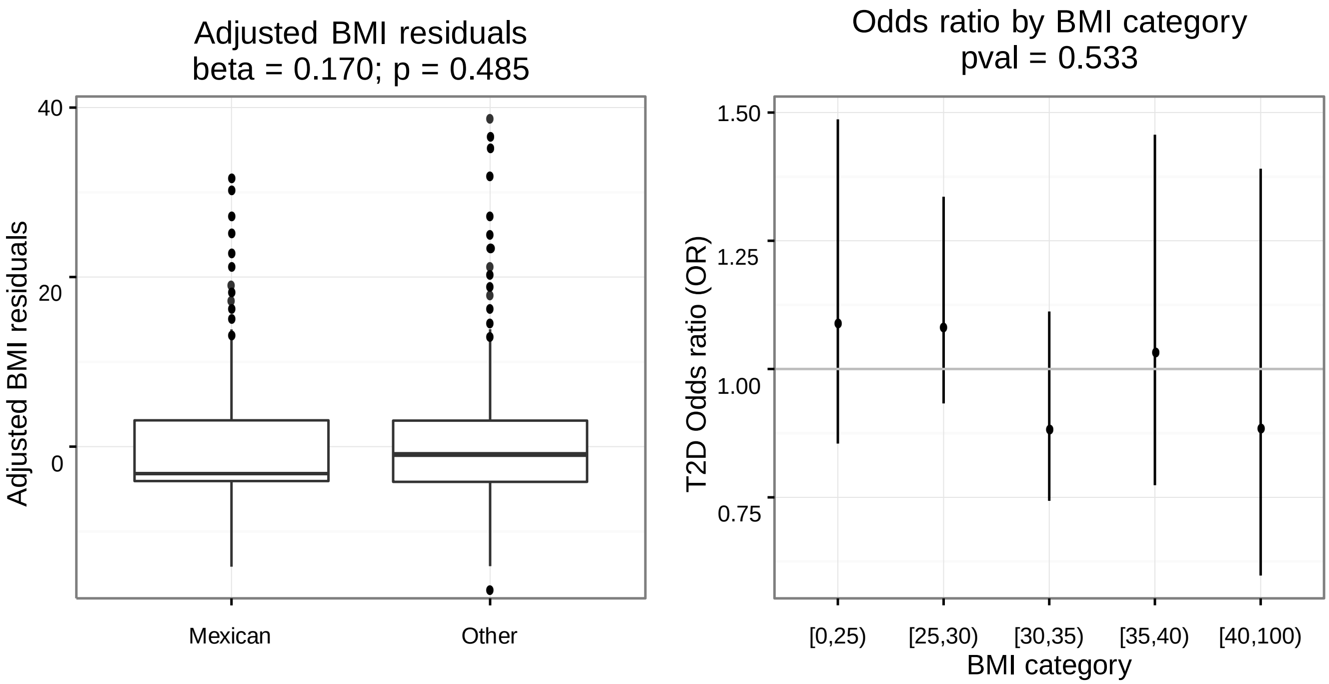

Figure S2: BMI analysis results.

The left panel shows a comparison of BMI between participants in the Mexican genetic analysis group ("Mexican") and participants in other groups ("Other"). BMI was regressed on age, sex, center, log sampling weight, and an indicator for being in the Mexican group, and includes three random effects for correlations due to block group, household, and genetic relatedness. The effect estimate and p-value for being in the Mexican group are given in the title. The y-axis is the residuals from this model adjusted for age, sex, center, and sampling weight only. The difference between the Mexican group and the Other group is not significant.

The right panel shows the DM odds ratio (OR) for rs75493593 in five different BMI categories from a pooled analysis that included age, sex, center, sampling weights, genetic PCs, and the three random effects. The p-value shown in the plot title is the p-value for interaction between BMI and the SNP effect on diabetes status. While Traurig et al found that the OR changed sign in larger BMI categories, there is not a clear trend in the

HCHS/SOL data, and the SNP×BMI category interaction term is not significant. We observed fewer individuals in the BMI>30 categories, which could explain non-significance of results in this analysis.

5 Fine Mapping - *SLC16A11*

We tried to fine map the region surrounding the SLC16A11 variants to identify the most associated Mexican SNP, and the most associated SNP in the combined cohort. However, none of the identified SNPs passed the multiple testing correction threshold.

Methods: Using imputation based on 1000G phase 1 reference panel, we attempted to fine-map the region surrounding the SLC16A11 variants. We defined the region as the 250K base pairs centered at rs13342232. Since the number of variants is high, we restricted our search to variants with at effN at least 250, where effN = 2N8MAF*(1-MAF)*oever approximates the count of the minor alleles of a variants. We considered two approaches: first we searched for the variant most associated with diabetes status in the Mexican group, and second, we searched for the variant most associated with diabetes status in the combined group. For each analysis, we calculated the effective number of independent variants in the region m using the simpleM method and determined a locus-wide significance threshold of 0.05/m. We report results from the same interaction model used for testing the SLC16A11 variants.

Results: The number of independent tests estimated in the analysis of the Mexican group was 419, leading to a locus-wide significance threshold of $1.1x10^{-4}$. The lead variant in the Mexican analysis group was an imputed indel rs33926848 (OR = 0.68, p-value = $2.2x10^{-4}$ of the minor allele, in the Mexican group), and was not statistically significant. For this variant, the p-values were high in the other genetic analysis groups (>0.16) and the estimated directions of associations were not consistent between groups. Accordingly, the meta-analyzed effect size of the minor allele was 0.9, p-value=0.07.

In the combined analysis of all groups, the estimated number of independent tests was 652, leading to a locus-wide significance threshold of 7.7×10^{-5}. The lead SNP was the imputed variant rs117787477 (OR =0.63, p-value= 2.8×10^{-3} of the minor allele, in the combined analysis). For this variant, the estimated directions of associations were the same across all groups, and the Mexican group had most significant association (p-value=0.01).

6 Diabetes status SNP Effects – Meta analysis Results

Table 1. Estimates of SNP effects on diabetes status for a meta-analysis of six HCHS/SOL background groups and for the Mexican group.

rsID	Risk/other allele	Meta-analysis OR [95% CI]	Meta P-value (one-sided)	Cochran's Q P-Values*	Mexican OR (one-sided P-value)
rs75493593	T/G	1.04 [0.92 - 1.17]	0.28	0.27	1.17 (0.025)
rs75418188	T/C	1.04 [0.92 – 1.17]	0.29	0.27	1.17 (0.026)
rs13342232	G/A	0.99 [0.89 – 1.10]	0.59	0.07	1.18 (0.019)
rs13342692	C/T	0.99 [0.89 – 1.10]	0.58	0.08	1.18 (0.019)
rs117767867	T/C	1.04 [0.92 – 1.17]	0.27	0.27	1.17 (0.024)

*Cochran's Q p-values for heterogeneity across all six groups.

Table 2. Summary results from one SNP from each of the two LD groups in HCHS/SOL.

rs13342232 *

group	Risk AF	N	OR [95% CI]
CentralAmerican	0.27	835	0.91 [0.67 – 1.23]
Mexican	0.30	2,879	1.18 [1.01 – 1.38]
SouthAmerican	0.27	563	0.80 [0.54 – 1.17]
Cuban	0.10	1,314	0.82 [0.58 – 1.17]
Dominican	0.20	740	0.90 [0.62 – 1.30]
PuertoRican	0.18	1,397	0.81 [0.62 – 1.05]
**Summary**	**0.23**	**7,728**	**0.99 [0.89 – 1.10]**

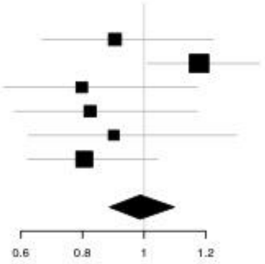

rs75493593 **

group	Risk AF	N	OR [95% CI]
CentralAmerican	0.22	835	0.90 [0.65 – 1.24]
Mexican	0.28	2,879	1.17 [1.00 – 1.37]
SouthAmerican	0.24	563	0.75 [0.49 – 1.14]
Cuban	0.04	1,314	0.88 [0.53 – 1.48]
Dominican	0.03	740	0.94 [0.42 – 2.14]
PuertoRican	0.07	1,397	0.89 [0.60 – 1.30]
**Summary**	**0.17**	**7,728**	**1.04 [0.92 – 1.17]**

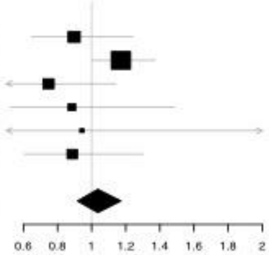

Supplement: Supplementary file 1 — Supplementary Information [file 41598_2018_35707_MOESM1_ESM.pdf]
